# Oral Verrucous Carcinoma Mimicking a Chronic Candidiasis: A Case Report

**DOI:** 10.1155/2012/190272

**Published:** 2012-06-06

**Authors:** Natália Galvão Garcia, Denise Tostes Oliveira, João Adolfo Costa Hanemann, Alessandro Antônio Costa Pereira

**Affiliations:** ^1^Area of Pathology, Department of Stomatology, Bauru School of Dentistry, University of São Paulo, Al. Dr. Octávio Pinheiro Brisolla 9-75, 17012-901, Bauru, SP, Brazil; ^2^Area of Stomatology, Department of Clinic and Surgery, Alfenas Federal University, Alfenas, MG, Brazil

## Abstract

Verrucous carcinoma has a special propensity to mimic benign lesions of the oral cavity. A case of the oral verrucous carcinoma in maxillary alveolar ridge, extending to buccal vestibule, cheek, and labial mucosa, which was diagnosed and initially treated as chronic candidiasis, is presented. Clinical, histopathological, and therapeutic considerations related to diagnosis of the verrucous carcinoma in the oral cavity are discussed.

## 1. Introduction

The verrucous carcinoma, a low-grade variant of squamous cell carcinoma, has been reported in the head and neck area, with predilection by oral cavity and larynx [1–4]. The clinical outcome of verrucous carcinoma often shows a local invasive pattern without any distant metastases and excellent prognosis [5–7].

The establishment of clinical or histopathological diagnosis of verrucous carcinoma in the oral cavity may be difficult to interpret. This difficulty has been attributed to the indolence of this tumor's grow, its benign histologic features, and the lack of awareness by the pathologist of the tumor's gross appearance [1, 4].

We present the case of a patient with oral verrucous carcinoma in maxillary alveolar ridge, which was diagnosed and initially treated as chronic candidiasis. Clinical, histopathological, and therapeutic considerations related to diagnosis of the verrucous carcinoma in the oral cavity are discussed.

## 2. Report of a Case

A 67-year-old female was referred for evaluation of verrucous white lesion in the oral cavity. There was no history of tobacco or alcohol consumption. According to the patient, the lesion was present for approximately 24 months and it had a slow growing in the last six months. She had been submitted to an incisional biopsy whose diagnosis was of chronic candidiasis. As a result, antifungal therapy (Fluconazol 150 mg/week) was provided during six months without any remission of the lesion.

**Figure 1:**
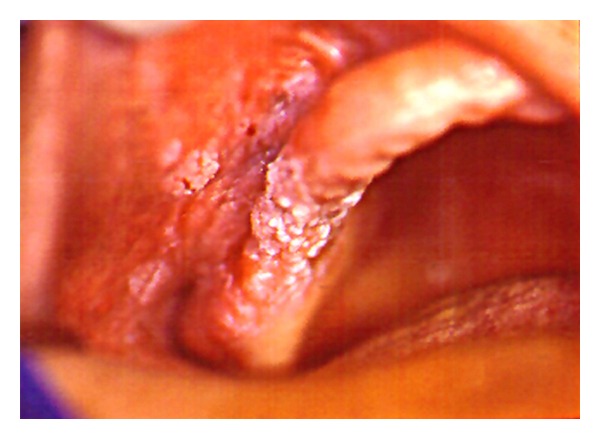
Verrucous white lesion in the right maxillary alveolar ridge, extending to buccal vestibule and cheek.

Clinical examination revealed the presence of a wide verrucous white lesion in the molars region of the maxillary alveolar ridge, extending to buccal vestibule, cheek, and labial mucosa (Figure 1). The submandibular lymph nodes were clinically normal.

Radiographs examination showed no infiltration of the maxillary bone. The differential diagnosis included verrucous leukoplakia, verrucous carcinoma, and squamous cell carcinoma. Then, two incisional biopsies were performed in different areas of the lesion and submitted to histopathological analysis.

**Figure 2:**
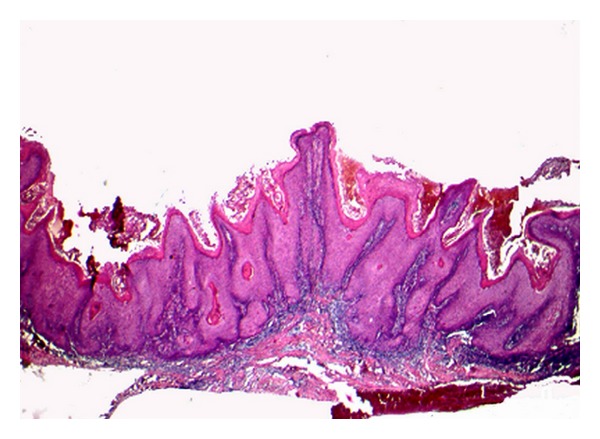
Histologic section showing stratified squamous epithelium with intense hyperplasia and hyperparakeratosis (hematoxylin and eosin stain, original magnification ×25).

**Figure 3:**
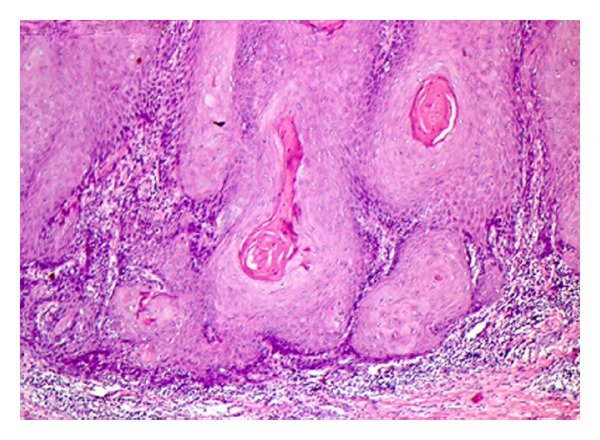
Histologic section showing low atypia degree, formation of keratin pearls, and, subjacent to epithelium, dense inflammatory infiltrate (hematoxylin and eosin stain, original magnification ×100).

**Figure 4:**
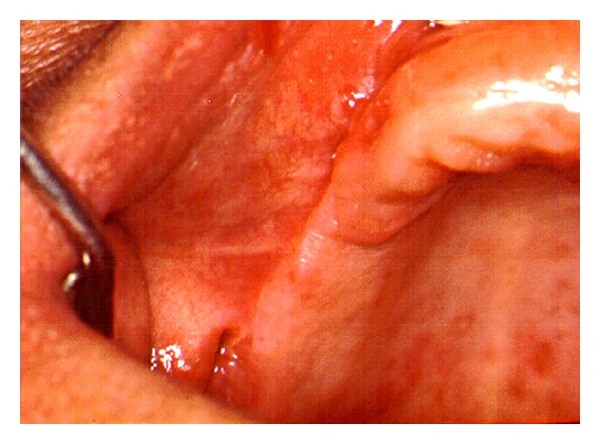
One-year follow-up photograph showing no local recurrence.

Microscopic features showed stratified squamous epithelium with intense hyperplasia, hyperparakeratosis, low atypia degree and formation of keratin pearls (Figure 2). The rete pegs were broad and appeared to “push” into the underlying tissues. Despite exaggerated rete pegs, the associated basement membrane appeared intact. Subjacent to epithelium, a moderate and dense inflammatory infiltrate was observed (Figure 3). The diagnosis established was verrucous carcinoma according to both clinical and histopathological patterns. The lesion was completely excised and the histopathologic examination of surgically resected specimen confirmed the diagnosis of verrucous carcinoma. The postoperative course was uneventful and a one-year followup did not detect local or regional recurrence (Figure 4).

## 3. Discussion

Generally, the verrucous carcinoma occurs in elderly and tumors of the tongue, gum and buccal mucosa are more common among females [2]. The etiological factor of verrucous carcinoma in the oral cavity is not completely established, although the lesion had been associated with tobacco use and human papilloma virus (HPV) [4, 8]. Eisenberg et al. found cytologic features generally associated with viral modification in 15 of the 17 cases of verrucous carcinoma in the oral cavity. However, in the present case, the patient said she never had used any kind of tobacco. Then, the oral verrucous carcinoma described above probably was associated with other carcinogenic factors instead of those related with tobacco. However, the primary causal factor associated to the development of this lesion was not clinically detected.

The diagnosis of the verrucous carcinoma may be difficult, requiring, in some cases, several biopsies. This lesion has a special propensity to mimic of benign tumors of the oral cavity. The clinical and histopathological differential diagnosis should include pseudoepitheliomatous hyperplasia, well-differentiated squamous cell carcinoma, chronic candidiasis, and condyloma accuminatum. The definitive diagnosis obviously requires concurrence between the clinician's appreciation of the tumor and the pathologist's identification of the microscopic criteria described by Ackerman [1, 6]. The histopathological diagnosis of verrucous carcinoma cannot be made if superficial biopsy is performed in the oral lesion. In the present case, the patient had been treated with systemic antifungal drug during six months because the initial diagnosis established was chronic candidiasis. Although oral verrucous carcinomas generally present slow growing, inappropriate therapy can contribute with local spread of the lesion.

The treatment of oral verrucous carcinomas, as in the reported case, consists of surgical excision without the need for lymph nodes dissection, because regional metastasis is rare [3, 4, 6, 9]. The radiotherapy combined with chemotherapy is the next most preferable treatment when surgery is not undertaken [9]. However, it has been reported approximately 30% of anaplastic transformation in verrucous carcinoma after radiation, inducing the occurrence of highly aggressive new neoplasm due to disregulation of the cell lines. Another explanation for anaplastic transformation is the possibility of hidden undifferentiated areas in verrucous carcinoma that start their proliferation later [10]. Although the lesion had an excellent prognosis, local recurrences may develop requiring re-excision [11]. Then, the one-year followup, as carried out in the reported case, is recommended.

Finally, it is important to reinforce that appropriate diagnosis of the verrucous carcinoma in the oral cavity can prevent the most extensive involvement of adjacent areas and/or wide surgical resection of the lesion including lymph nodes dissection as recommended for some cases of oral well-differentiated squamous cell carcinoma.
